# 

**DOI:** 10.1038/sj.bjc.6600380

**Published:** 2002-01-02


line was determined during a 3-day continuous incubation. There were marked differences in sensitivity to each drug (RCC39, RCC42, RCC45 $EC_{50}$ for 5-FU 0.8, 3.4, 0.5 mM and for SN-38 240, 39, 17 ng/mL respectively). $EC_{25}$ concentrations were derived from these model fits, and cells were then treated at these concentrations for 2 sequential 3-day periods, with: SN-38 alone d1-6; 5-FU alone d1-6; SN-38 + 5-FU in combination d1-6; SN-38 d1-3 followed by 5-FU d4-6; 5FU d1-3 followed by SN-38 d4-6. The use of both drugs in combination, or 5-FU followed by SN-38, produced no better than additive effects in any cell line. However, in RCC39 and RCC42, the sequence of SN-38 followed by 5-FU showed marked synergy, with % viabilities on day 6 of only 20% and 17% respectively ($p<0.01$ for both cell lines, compared to the expected effect). Synergy was not found with RCC45. In RCC39 and RCC42, 5-FU exposure resulted in characteristic increases in TS protein by day 3, but this increase was not observed in either cell line when SN-38 was given prior to 5-FU.

These data suggest that SN-38 and 5-FU may act synergistically in renal cancers, and that sequence is critical in this interaction. This effect may be mediated via TS.

As both drugs show activity in this setting, these data support the investigation of this sequence in a clinical trial.

**P230**

**NU:UB 199: A NEW COLON SELECTIVE AGENT THAT TARGETS TOPOISOMERASE I AND THE β-ISOFORM OF TOPOISOMERASE II.**

L Young[1]*, D J Mincher[1], A Turnbull[1], G Kay[1], S Pettersson[1], M C Bibby[2], J A Double[2], [1]Anticancer Drug Design and Delivery Research Group, School of Life Sciences, Napier University, Edinburgh, EH10 5DT, UK; [2]Cancer Research Unit, University of Bradford, BD7 1DP, Bradford, UK.

The anthraquinone conjugate of the unnatural amino acid (S)-cyclohexylalanine, NU:UB 199, is a dual inhibitor of topoisomerase I and II enzymes with a novel pattern of inhibition. NU:UB 199 stimulated topo I-induced DNA cleavage (44% nicked pBR322 plasmid) *in vitro*. In common with camptothecin, this conjugate also stabilises covalent topoisomerase I-DNA cleavable complexes in intact cells and thus behaves as a topo I poison. The extent of immunoband depletion of topo I in human HL60 cells by NU:UB 199 at 100μM was comparable to that obtained with camptothecin at 50μM when applied to the incubation medium for 45 minutes. Additionally, the conjugate is a catalytic inhibitor of topo II; inhibition of topo II-mediated relaxation of pBR322 plasmid DNA *in vitro* is complete at 1μM. In HL60 cells, immunoband depletion (complete at 200μM) of topo IIβ, but not the α-isoform, was observed, suggesting preferential targeting of the β-isoform. NU:UB 199 is active in the low μM range across the NCI 60-cell line panel and shows exceptional selectivity for the cell lines of the colon sub-panel. Furthermore, this conjugate is active in the MAC15A murine adenocarcinoma tumour model that is refractory to standard agents. The pre-clinical data indicates that NU:UB199 is a promising new anti-tumour agent that targets both topo I and topo II and does not suffer from the problems of structural lability and short-lived drug-stabilised DNA-topo cleavable complexes normally associated with the camptothecins.

**P231**

**DT-DIAPHORASE AND CYTOCHROME P 450 REDUCTASE OVEREXPRESSION SENSITISES HUMAN BREAST CANCER CELL LINES TO MITOMYCIN C TREATMENT BOTH *IN VITRO* AND *IN VIVO*.**

Kaye J. Williams*[1], Adam V. Patterson[2], Brian A. Telfer[1], Rachel L. Cowen[1], Mohammed Jaffar[1], Ian J. Stratford[1] School of Pharmacy and Pharmaceutical Sciences, University of Manchester, Manchester, M13 9PL[1]; Auckland Cancer Society Research Centre, University of Auckland, Auckland 1000, NZ[2].

The refractive nature of hypoxic tumour cells to most forms of cancer therapeutics makes this population an attractive target for anti-cancer drug design. We are currently developing indolequinones as bioreductive drugs for use in cancer chemotherapy. These compounds require enzymatic reduction to yield the cytotoxic moiety. However, the expression of the one- and two-electron reducing enzymes required for drug activation is heterogeneous in human malignancies. To overcome the potential variability in reductive-activation we aim to exploit specific enzyme: bioreductive drug combinations in an enzyme – directed gene therapy approach. We have investigated the role of two key reducing enzymes, DT-diaphorase (DTD; two-electron) and cytochrome c P450 reductase (P450R; one electron) in the cytotoxicity of the prototype bioreductive drug, mitomycin C (MMC). Stable cell lines were generated from three breast carcinoma cell lines (T47D, MDA 231 and MDA 468) to overexpress either DTD or P450R. The level of enzyme activity achieved varied between 200-1000 fold increase in activity compared to wild-type cells for DTD and 20-60 fold increase for P450R. Both DTD and P450R overexpression sensitised cells to MMC in aerobic conditions. The most profound effect was seen in the MDA 468 cells, where the $IC_{50}$ value for MMC was reduced 27-fold by DTD over-expression. P450R over-expression yielded a five-fold reduction in $IC_{50}$ in this cell line. No specific increase in cytotoxicity was seen when the clones were exposed to drug under anoxic conditions. To analyse whether this sensitisation was maintained *in vivo*, MDA468 wildtype cells and the DTD and P450R over-expressing clones were established as xenografts in nude mice. The fold-increase in enzyme activity in the DTD and P450R clones relative to wildtype was maintained, but the absolute levels of activity were markedly reduced in all tumour types compared to that seen *in vitro*. In keeping with our *in vitro* findings, the MMC sensitisation achieved by DTD over-expression was greater than that by P450R over-expression *in vivo*, with T/C (treated tumour volume/control tumour volume x 100) of 18% for DTD and 61% for P450R at the time of removal of the first control tumour. We now aim to evaluate adenoviral delivery of DTD and P450R as a means of sensitising established human xenografts to MMC, against which we can compare the efficacy of our novel indolequinone drugs in future studies.

**P232**

**VESICLE-POLYMER HYBRID GENE DELIVERY SYSTEMS IMPROVE IN VIVO EFFICACY AND REDUCE TOXICITY OF PEI BASED SYSTEMS**

Brownlie A[1,2], Uchegbu IF[2], Schätzlein AG[1*]

[1]Department of Medical Oncology, Cancer Research UK Beatson Laboratories, University of Glasgow, Garscube Estate, Glasgow G61 1BD

[2]Department of Pharmaceutical Sciences, University of Strathclyde, 27 Taylor St, Glasgow G4 0NR.

**Introduction:** For most gene therapy approaches to cancer it is crucial to transfect the therapeutic gene into most if not all the transformed cells. Although different types of bystander effects provide some leverage in improving the efficacy of treatments the specific, efficient, and safe delivery of the therapeutic gene remains an obstacle which hampers the progress of these novel therapies into the clinic[1].

Polyethylenimine (PEI) polymers have proven versatile and efficient synthetic gene delivery systems, but despite its efficacy PEI has some disadvantages, namely the bias of biodistribution to the lung and its significant toxicity in vitro and in vivo.

Using a modular modification strategy we have created a vesicle/polymer hybrid system with improved performance and safety profile, particular in vivo.

**Methods:** 4 PEI variants were produced: namely palmitoyl-PEI (P-PEI), palmitoyl-polyethylene glycol-PEI (PP-PEI), quaternary ammonium-PEI (Q-PEI), and quaternary ammonium palmitoyl-PEI (QP-PEI). The chemistry and physicochemistry of the polymers and the derivatives were characterised (elemental analysis, 1H-, 13C- NMR, dynamic and static light scattering, electron microscopy, zeta potential). The biological testing involved assays of cytotoxicity (MTT), serum/plasma interaction, haemolysis, and erythrocyte aggregation. Efficiency of transgene expression was assessed using b-galactosidase (in vitro) or GFP (in vivo, mouse model) using immunohistochemistry.

**Results:** Changes of key physicochemical properties such as charge, nitrogen density, and self-aggregation were linked to the biological properties of these systems. All polymers condensed DNA at a polymer, DNA weight ratio of 2.5: 1 or less. The haemolytic activity of the polymers was very low and all of the modified polymers proofed to be less cytotoxic than the parent polymer. Although the parent polymer PEI was superior to the modified polymers as a transfection agent in vitro the hybrid systems were superior in

 © 2002 Cancer Research UK

vivo with high expression in the liver; the amphiphilic polymer variants and followed the trend QP-PEI > P-PEI > PEI > Q-PEI.

**Conclusion:** Polymer/vesicle hybrid type gene delivery systems maybe used to combine and exploit the complementary qualities of both, lipoplexes and polyplexes, in one system. It is known that good gene expression in vitro does not in anyway predict good expression levels in vivo. Here we demonstrate that the opposite is also true, i.e. the lack of expression in vitro does not predict for lack of expression in vivo.

**References** Schätzlein AG. Non-viral vectors in cancer gene therapy: principles and progress. *Anti-cancer Drugs* 2001; **12:** 275-304.

### P233

### TARGETING OF NANOCONJUGATES TO SOLID TUMOURS

Corrihons F[,2*], Moore BD[2], McCusker K[3], Kocienski P[3], Schatzlein AG[1]
[1]Department of Medical Oncology, Cancer Research UK Beatson Laboratories, University of Glasgow, Garscube Estate, Glasgow G61 1BD
[2]Department of Pharmaceutical Sciences, University of Strathclyde, 27 Taylor St, Glasgow G4 0NR.
3Department of Chemistry, University of Leeds, Leeds,LS2 9JT

**Background:** While particulate drug carriers such as liposomes have proven to be clinically useful in various cancers targeted delivery of drugs to solid tumours remains a considerable challenge. The main barriers experienced when targeting to the tumour are closely related to the interplay between tumour growth and tumour vascularisation. Tumour induced blood vessels (neovascularisation) have a leaky architecture which, in combination with compression of surrounding tissue, leads to an increased interstitial pressure and changes the flow of biological fluids with in the tumour mass. This limits convective, size-independent transport, and distribution of molecules and carriers throughout the tumour rather depends on diffusion, with the flux correlating inversely with the molecular weight of the drug/carrier. To address this problem we have developed low molecular weight conjugates (< 3kD), so called nanoconjugates, of a small drug or toxin and a targeting ligand coupled through a (cleavable) linker group. Nanoconjugates offer potential advantages for tumour specific delivery in diffusion-limited situations.

**Method:** Phage display was used to identify small peptides that target the transferrin receptor (TfR), which is frequently overexpressed in tumours. A library of phage expressing a cyclic nona-peptide was panned against TfR. The panning procedure was monitored using eluted phage counts and surface plasmon resonance. Another ligand tested was based on cyclic integrin binding peptides targeting the αVβ3 integrins. Various conjugates of the targeting peptides carrying fluorescent probes or a model drug, doxorubicin, were prepared, purified and characterised. Some of the doxorubicin conjugates were prepared using a intracellular cleavable peptide linker between targeting moiety and drug. Biological properties were tested using MTT cytotoxicity assays, flow cytometry and confocal microscopy.

**Results:** In the panning process a peptide ligand, which mediated a 30-50-fold increased receptor specific cellular uptake of the phage, was identified. The ligand targeted nanoconjugates–in contrast to the free drug–are being taken up by receptor mediated endocytosis. This appeared to be specific (TfR) as it could be inhibited with excess substrate. Uptake was increased in receptor positive cell lines after 2h incubation, but this advantage was lost after longer exposure (αVβ3). Cytotoxicity was significantly lower that that of free drug (Dox) with and with our the cleavable linker. Experiments to determine whether these targeted nanoconjugates may be useful for the targeting of tumours in vivo are on-going.

### P234

### COMPUTER AIDED OPTIMISATION OF SYNTHETIC GENE DELIVERY AGENTS USING PPI DENDRIMERS

Bernd H. Zinselmeyer[1,2,*], Simon P. Mackay[2], Ijeoma F. Uchegbu[2], Andreas G. Schatzlein[1]
[1]Department of Medical Oncology, Cancer Research UK Beatson Laboratories, University of Glasgow, Glasgow G61 1BD
[2]Department of Pharmaceutical Sciences, University of Strathclyde, 27 Taylor St, Glasgow G4 0NR.

The issue of gene delivery has recently been identified as the bottleneck for human gene therapy in general [1]. For cancer gene therapy it is namely the lack of transfection efficiency and specificity, of synthetic gene delivery agents which hamper transfer of new therapeutic strategies into the clinic [2]. Viral vectors, while more efficient, have raised serious safety concerns.

While numerous compounds have been tested as potential delivery agents this approach was historically based on trial and error. Rational design of molecules which show optimum interaction with DNA would greatly accelerate the development of more efficient systems.

The use of dendrimers as gene delivery agents has been largely focused on the use of the polyamidoamine molecules and although polypropylenimine dendrimers contain 100% protonable nitrogen and bind DNA they have been relatively poorly studied as gene transfer agents. Here we demonstrate how insights from computer based molecular modelling can be used to select suitable molecules from a series of polypropylenimine dendrimers.

**Methods**: DNA binding was evaluated by measuring the reduced fluorescence of ethidium bromide and molecular modelling of dendrimer – DNA complexes was also carried out. Cell cytotoxicity was evaluated against the A431 cell line using the MTT assay. In vitro transfection was evaluated against the A431 cell line using the β-galactosidase reporter gene and DOTAP, served as a positive control.

**Results**: Molecular modelling revealed that the number of dendrimer – DNA contact points per dendrimer and the simulated dendrimer - DNA intermolecular energies increase with dendrimer generation. DNA condensation (measured by the exclusion of ethidium bromide), and experimentally determined dendrimer - DNA positive zeta potential also increased with dendrimer generation. Cell cytotoxicity was largely but not exclusively generation dependant and cytotoxicity followed the trend DAB 64 > DAB 32 > DAB 16 > DOTAP > DAB 4 > DAB 8 while transfection efficacy followed the trend DAB 8 = DOTAP = DAB 16 > DAB 4 > DAB 32 = DAB 64.

**Conclusion** The generation 2 polypropylenimine dendrimer combines a sufficient level of DNA binding with a low level of cell cytotoxicity to give it optimum gene transfer activity in vitro.

1. Anderson WF. Human gene therapy. Nature 1998; 392: 25-30.
2. Schätzlein AG. Non-viral vectors in cancer gene therapy: Principles and progress. Anti-cancer Drugs 2001; 12: 275-304.

### P235

### *IN VITRO* AND *IN VIVO* COMBINED GENE & IMMUNOTHERAPY VECTORS IN PROSTATE CANCER

James G. Young[1], Nicola K. Green[1], Peter F. Searle[1], Alan Doherty[2], D. Michael A. Wallace[2], Lawrence S. Young[1], Nicholas D. James[1]*.
(1). CRC Institute for Cancer Studies, Vincent Drive, Edgbaston, Birmingham B15 2TA. (2). University Hospital Birmingham.

**Introduction and Objectives:** A novel gene therapy system has been developed using adenoviruses encoding E. Coli nitroreductase (NR), which converts the prodrug CB1954 into a potent alkylating agent. To elicit additional systemic antitumour effects single and combination adenoviral vectors expressing NR and mouse granulocyte-monocyte colony stimulating factor (mGMCSF) have also been generated. The syngeneic TRansgenic Adenocarcinoma Mouse Prostate (TRAMP) model of prostate cancer was utilised both *in vitro* and *in vivo* to characterise the effects of these vectors in prostate cancer. **Methods:** UV microscopy and FACS analysis were used to assay infectivity of the cell lines by adenoviruses *in vitro* with a vector expressing the marker gene Green Fluorescent Protein (GFP). A □-galactosidase expressing vector was used to assay infectivity and transgene expression levels *in vivo.* Subcutaneous flank tumours were established in syngeneic C57/Bl6 mice for *in vivo* studies of both intratumoural injection and *ex-vivo* gene therapy. **Results:** Approximately 100% of TRAMP cells were infected at an adenoviral multiplicity of infection (MOI) of 100. *In vitro* enhancement of toxicity to CB1954 was similar in cells infected with the dual virus or a virus expressing NR on its own. Studies of established subcutaneous TRAMP C2 tumours injected with Ad5-β-Galactosidase showed 1000 fold increases in transgene expression compared with mock infections. Injection of the dual expressing vector Ad5-mGMCSF-NR produced a survival benefit in animals with TRAMP C2 tumours, but no additional benefit in survival was produced by injection of CB1954. *Ex-vivo* gene therapy of TRAMP C2 cells with Ad5-mGMCSF ablated tumour development compared with control adenovirus. **Conclusions:** Efficient

© 2002 Cancer Research UK

infection and expression of transgenes from adenoviral gene therapy vectors has been shown in the TRAMP model. Subcutaneous tumours grown in syngeneic mice respond to intratumoural Ad5-mGMCSF/NR but addition of prodrug conferred no additional survival advantage. Ex-vivo treatment of TRAMP C2 cells with Ad5-mGMCSF ablates tumourigenesis, suggesting that mGMCSF is the therapeutically active transgene.

**P236**

**COMBINING ONCOLYTIC AND VDEPT APPROACHES TO PROSTATE CANCER**

James G. Young[1], Peter F. Searle[1], Alan Doherty[2], D. Michael A. Wallace[2], Lawrence S. Young[1], Nicholas D. James[1]*.
(1). CRC Institute for Cancer Studies, Vincent Drive, Edgbaston, Birmingham B15 2TA. (2). University Hospital Birmingham

**Introduction and Objectives:** An adenovirus *dl1520*, which selectively replicates in & kills cancer cells, is produced by deletion of the adenoviral E1B sequence. Variants of *dl1520* containing the gene for the prodrug-converting enzyme *E. Coli* nitroreductase (NR) were generated in an attempt to enhance cancer cell killing. The transgene is placed under the control of a prostate specific transcriptional regulatory region. **Methods:** The gene for NR was cloned downstream from a 632bp PSA promoter sequence and a single 1469bp PSA enhancer sequence into the E1 region of the adenoviral genome. Replication of the virus was analysed by dot blotting of viral DNA preparations. LNCaP cells were infected with a similar marker virus encoding GFP and transgene expression analysed by UV microscopy and FACS analysis. NR expression was analysed by Western blotting of protein lysates of LNCaP cells infected with adenovirus encoding NR **Results:** Replication of the adenoviral vector within LNCaP cells was demonstrated on dot blot analysis of viral DNA preparations. Infection of LNCaP cells with virus encoding GFP demonstrated high levels of transgene expression by both UV microscopy and FACS analysis. Western blot analysis confirmed rising NR expression levels over a 5-day time course. Infection of LNCaP cells with NR encoding adenovirus and addition of the prodrug CB1954 produced additive cell killing. **Conclusions:** Novel adenoviruses capable of replicating and expressing marker and therapeutic transgenes in the prostate cancer cell line LNCaP have been produced. Transgene expression levels from the PSA TRR can be increased by viral replication. Additive cell killing effects have been achieved using this vector in its capacity as gene therapy vector and viral oncolytic agent.

**P237**

**NOVEL LIGANDS FOR THREE– AND FOUR–STRANDED DNA STRUCTURES: SYNTHESIS, BIOPHYSICS AND CYTOTOXICTY.**

Victoria A. Phillips,*[1] Sharon A. Jennings,[1,2] Terence C. Jenkins,[2] Roger M. Phillips,[3] Richard T. Wheelhouse.[1]
[1]School of Pharmacy, [2]YCR Laboratory of Drug Design and [3]Cancer Research Unit, The University of Bradford, BRADFORD, BD7 1DP, UK.

Three-and four-stranded DNA structures are important targets in drug design. Antigene strategies for protein down-regulation require adjuvant ligands to stabilise triplex structures; four-stranded DNA is implicated in c-*myc* expression and the abnormal genomic DNA of Werner's, Bloom's and Fragile-X syndromes, besides its role in the regulation of telomerase. Ligands with precisely-defined structural selectivity could therefore have a range of potential therapeutic applications. Furthermore, the avoidance of undesirable cytotoxic effects dictates that any competing affinity for duplex structures must be eliminated in the course of the design.

Pyrimidines bearing (ω-aminoalkyl)aryl substituents (e.g. **1**) are intriguing ligands for high-order DNA structures. Preliminary studies[1] indicate that thioether-linked compounds are remarkable stabilisers of triplex DNA, whilst minor alteration of the functional groups in the linker switches the structural preference toward four-stranded DNA structures.

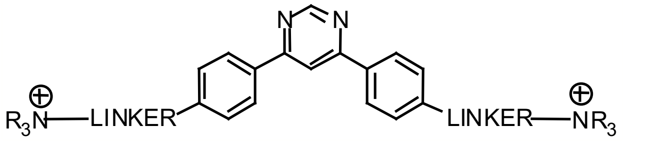

**1**

Novel 4,6-diarylpyrimidines and related chromophores have been designed and synthesised to explore how the linker functional groups and length, the disposition of charges and amine p$K_a$ influence the SAR for interaction with relevant nucleic acid secondary structures. Details of the synthesis, biophysical studies and *in vitro* cytotoxicity will be presented.

(1) Wheelhouse *et al*., 2001, *Clin, Cancer Res.* **7** 711 Suppl. S. Murphy *et al*., 2001, *Br. J. Cancer* **85** S1 92.

**P238**

**NOVEL FUNCTIONALISED INHIBITORS OF O6-ALKYLGUANINE–DNA ALKYLTRANSFERASE.**

Dimitrios Pletsas,*[1,2] Anna Nicolaou,[1] Vassiliki Pletsa,[3] Soterios A. Kyrtopoulos,[3] Michael C. Bibby,[2] Richard T. Wheelhouse.[1]
[1]School of Pharmacy and [2]Cancer Research Unit, The University of Bradford, BRADFORD, BD7 1DP, UK. [3]National Hellenic Research Foundation, 48, Vas. Constantinou Avenue, 11635 Athens, Greece.

Many tumour cell lines develop resistance towards alkylating chemotherapeutic agents due to the active status of the DNA repair protein O6-alkylguanine-DNA alkyltransferase (ATase). This protein recognises and repairs the alkylated lesions formed at the O6 position of guanine bases in DNA, through which the cytotoxic effect would otherwise be derived. Hitherto, ATase inhibitors have been designed to covalently modify the active site of the protein, e.g. benzylguanine. Herein we present the design, synthesis and preliminary evaluation of a different kind of substrate analogue that may interact non-covalently with the ATase protein.

Careful examination of X-ray crystal structures of the active site of the human form of the repair protein – in its native form and covalently modified by known inactivators of the protein – coupled with consideration of the reaction mechanism of the repair process has allowed the design of guanine derivatives which might bind in the active site by electrostatic and hydrogen bonding interactions. Furthermore, these novel derivatives bear acidic or basic functional groups which might interfere with catalysis of the DNA repair reaction. Such substrate analogues may be useful probes for crystallographic studies of purine-protein complexes and have application to the potentiation of cytotoxic chemotherapy.

Details of the design and synthesis of novel 6-substituted purines will be presented, accompanied by preliminary data on their ability to bind and inhibit the ATase protein.

**P239**

**A POTENTIALLY TUMOUR SPECIFIC TARGETED RADIOTHERAPY /GENE THERAPY STRATEGY FOR TREATMENT OF MALIGNANT DISEASE**

M Boyd[1], *SC Ross[1], P Welsh[2], G Akubani[2], MJ Zalutsky[3], WN Keith[3] and RJ Mairs[1].
1. Dpt. of Radiation Oncology, Cancer Research UK Beatson Labs, Glasgow, G61 1BD
2. Dpt of Radiology, Duke University Medical Centre, North Carolina, USA, 27710.
3. Dpt or medical Oncology, Cancer Research UK Beatson Labs, Glasgow, G61 1BD

Aims: We have developed a novel targeted radiotherapy/gene therapy strategy for treatment of malignant disease utilising introduction of the Noradrenaline transporter gene (NAT) into malignant cells. We introduced NAT into non-NAT expressing UVW glioma cells and endowed the cells with the ability to achieved high levels of [$1^{31}$I]MIBG and [$^{211}$At]MABG uptake and dose-dependent cell kill. A requirement for cancer gene therapy, is tumour specific expression of therapeutic transgenes. We investigated potentially tumour specific promoters, (the human telomerase promoters

© 2002 Cancer Research UK

hTERC and hTERT), for their capacity to express NAT in UVW glioma cells at levels sufficient for therapeutic effect following radionuclide administration.
Methods: UVW cells were stably transfected with the NAT gene under the control of the RSV, hTERT or hTERC promoter elements. Gene expression levels were determined by MIBG uptake. Cytotoxicity was assessed in multicellular tumour spheroids via clonogenic survival and regrowth delay after administration of [$^{211}$At]-astatinated benzylguanidine ([$^{211}$At]MABG) and [$^{131}$I]MIBG.
Results and Conclusions: UVW cells transfected with RSV/NAT, hTERC/NAT and hTERT/NAT were endowed with the capacity for active uptake of [$^{131}$I]MIBG and [$^{211}$At]MABG. NAT gene expression via the hTERC and hTERT promoters resulted in uptake levels 70% and 60% of that achieved by the RSV promoter control. We observed dose dependant cell kill of clonogens derived from [$^{131}$I]MIBG-treated spheroids. Reduction in surviving fraction was related to promoter activity. Administration of [$^{211}$At]MABG resulted in spheroid sterilisation at doses 1000 times lower than those required using [$^{131}$I]MIBG. All NAT transfected cells, regardless of the promoter employed, were sterilised by the same dosage of [$^{211}$At]MABG. These results indicate that the human telomerase promoters are excellent candidates for tumour-specific transgene expression and that NAT gene transfer in combination [$^{211}$At]MABG treatment is a potent anti-cancer strategy.

**P240**
**VALIDATION OF CYTOCHROME P450 CYP1B1 AS A TARGET FOR CANCER THERAPEUTICS,**

ML Greer[1*], GI Murray[2], GD Wilson[1], LH Patterson[3], SA Everett[1], [1]Gray Cancer Institute, PO Box 100, Mount Vernon Hospital, Northwood, Middlesex HA6 2JR, UK, [2]Department of Pathology, University of Aberdeen, Aberdeen, AB25 2ZD, UK, [3]The School of Pharmacy, University of London, 29/39 Brunswick Square, Bloomsbury, London WC1N 1AX, UK

CYP1B1 is a cytochrome P450 mono-oxygenase which is consistently over-expressed in a range of human tumours (reviewed by GI Murray et al., Annu Rev Pharmacol Toxicol. 2001;41:297-316). As part of the target validation process CYP1B1 protein expression has been characterised in clinical head and neck squamous cell carcinomas (HNSCCs) and in a series of human tumour xenografts from low passage head and neck cancer cell lines. The latter to be evaluated as a clinical model to test a novel CYP1B1/prodrug combination designed to target the delivery of the radiosensitiser/cytotoxin nitric oxide to tumours.
Immunhistochemistry was performed using a monoclonal antibody specific for CYP1B1 which did not react with other cytochrome P450s including CYP1A1 and CYP1A2 (McFadyen et al., J Histochem Cytochem. 1999;47:1457-1464). HNSCCs taken from a range of anatomical sites were of variable grade (n = 23). The intensity of immunoreactivity was assessed on a semi-quantitative scale as strong (3+), moderate (2+), weak (1+), negative (-). The majority of tumours showed positive immunoreactivity for CYP1B1 but both inter- and intra-patient variability in CYP1B1 protein expression was evident. Only three (13%) of tumours did not exhibit any immunoreactivity for CYP1B1, two tumours (9%) showed weak CYP1B1 immunoreactivity, six (26%) tumours displayed moderate immunoreactivity while twelve (52%) showed strong CYP1B1 immunoreactivity. In each case CYP1B1 expression was localised in the cytoplasm of the tumour cells and was absent from the associated normal stromal tissue. Spectral image analysis technology developed at the Gray Cancer Institute, which exploits the characteristic absorptions of individual stains (to distinguish stained from non-stained areas or co-localisation of stains), was used to confirm tumour-specific localization of CYP1B1. Similar patterns of expression have been observed previously in a range of malignancies and emphasizes the importance of CYP1B1 as a target for cancer therapeutics.
Previously, the inability to identify suitable experimental tumour models, which constitutively express CYP1B1 (with the frequency and intensity observed in clinical tumours), has proven a hinderance to understanding the underlying mechanisms of CYP1B1 regulation. In this study human tumour xenografts from low passage head and neck cell lines (including the UT16A, UT24A, UT15, UT33) have been found to contain the CYP1B1 phenotype. The UT16A and UT24 xenografts in particular exhibited strong CYP1B1 protein expression compared to many human tumour xenografts derived from established cell lines.
In conclusion, HNSCC expresses the CYP1B1 protein with high frequency (n = 23, 90%). A suitable *in vivo* model for studying both CYP1B1 regulation and testing CYP1B1-activated cancer therapeutics has been identified.
*This work is supported by Cancer Research UK.*

**P241**
**SUICIDE GENE THERAPY USING CYTOSINE DEAMINASE/5-FLUCYTOSINE SYSTEM IN A RAT PROSTATE CANCER MODEL.**

R Greenhalgh*, D Cook, I A Clarke, A G Dalgleish, H Pandha.
Dept. Oncology, St George's Hospital Medical School, Cranmer terrace, London, SW17 0RE

Introduction:
New treatments are needed for patients with hormone refractory prostate cancer. We have investigated its effectiveness of the CD/5-FC suicide gene therapy system in a rat prostate cancer model.
Method:
PA3 cells were stablely transfected with cytosine deaminase using a double-copy recombinant retrovirus under the control of the ERBB2 promotor. Transfection was confirmed using immunohistochemistry.
Cell cytotoxicity with 5-FC was assessed using the MTS assay.
Apoptosis was assessed using the commercially available Apoptag kit.
Bystander effect was assessed by plating cells at different concentrations of wild type and transfected cells. Cell viability was assessed using the MTS assay.
Results:
Cytosine deaminase expression was confirmed by immunohistochemistry.
Six days treatment with 10ug/ml 5-FC resulted in 40% cell death in the PCD cells compared to only 5% on the wild-type.
Apoptosis was shown in 30% of PCD cells compared to 0% in PA3-WT after 6 days treatment with 5-FC.
Conclusions:
In our studies cell death by apoptosis can be shown in transfected cells in vitro. We have shown a significant bystander effect in vitro and are currently using this system in an animal model.

**P242**
**CYTOSOLIC HUMAN NOS II ACTIVITY IN MDA 231 BREAST TUMOUR TRANSFECTED CELLS ENHANCES SR4233 ACTIVATION AND TOXICITY.**

Edwin Chinje*, Jian Feng, Sanjeev Paul Sharma, Natasha Wind, Rachel Cowen and Ian Stratford. Experimental Oncology Group, School of Pharmacy, University of Manchester, Oxford Road, Manchester, M13 9PL, UK.

SR4233 (tirapazamine, TPZ) is a lead bioreductive drug currently in clinical trials. It is selectively toxic to hypoxic cells commonly found in solid tumours and toxicity results from the intracellular metabolism of the drug to a highly toxic radical under hypoxia. Nitric oxide synthase (NOS) is an endogenously up-regulated enzyme that could provide tumour selective metabolism of chemotherapeutic prodrugs. Significantly, elevated levels have been found in several neoplastic tissues. Its dimeric nature provides two distinct catalytic domains (a reductase and an oxygenase) that will bioactivate a broad repertoire of chemotherapeutic agents including TPZ. The enzyme is a haem-based protein similar to members of the cytochrome P450 family and the reductase domain shares a high degree of sequence homology with cytochrome P450 reductase (P450R). However, unlike the former two enzymes, NOS II (inducible NOS) is soluble and exclusively expressed within the cytoplasm. Since, some reports have suggested that only metabolism of TPZ in the nucleus is likely to contribute to DNA damage [*Evans JW et al., 1998, Cancer Res. 58: 2098- 2101*], we have exploited this differential cellular localization to evaluate the contributions of cytosolic metabolism of TPZ to its overall toxicity. To achieve this, we have constitutively over-expressed NOS II by employing an optimised expression vector in which the human elongation factor 1α promoter produces a bicistronic message containing the genes for human NOS II and puromycin resistance. Following successful transfection of the human breast cell line,

© 2002 Cancer Research UK

MDA231, clones have been selected that stably express varying levels NOS II activity.
NOS reductase activity as measured by the NADPH-dependent reduction of cytochrome c ranged from 7.5–22.0 nmol cyt. c reduced/min/mg. The highest activity represented about 6-fold increase over parental activity. Similarly, the catalytic activity of the oxygenase domain, measured by the conversion of $^{14}$C-labelled L-arginine to L-citrulline, ranged from 20–66 pmol citrulline / min/mg and the highest activity was about 110-fold over parental cell activity. NADPH-dependent metabolism of TPZ to the stable two-electron metabolite, SR4317, was determined by HPLC analysis. Rate of metabolism was enhanced by the increase in NOS II expression. In addition, direct detection of the TPZ radical formation by the dihydrorhodamine (DHR) 123 assay, correlated with HPLC metabolism data ($r^2$ = 0.92). DT-diaphorase activity (can metabolise TPZ in a single 2-electron step by-passing the free radical) did not alter as a result of the clonal process. Sensitivity to TPZ following 3 hr hypoxic exposure also increased with elevated NOS II levels. Taken collectively, our data shows that cytosolic NOS II may contribute significantly to the bioactivation of tirapazamine.

**P243**

**CHARACTERISATION AND QUANTIFICATION OF DNA ADDUCTS FORMED BY THE PLATINUM DRUG BBR 3464.**

**TRL John*[1], CJ Ottely[2], DG Pearson[2], MJ Tilby[1].**
[1]Cancer Research Unit, Medical School, University of Newcastle upon Tyne, NE2 4HH. [2] Department of Geological Sciences, University of Durham, Science Laboratories, South Road, Durham, DH1 3LE.

**Background** The development of cisplatin resistance has seen the need for novel anti-cancer agents that can overcome this resistance. BBR 3464 is the first trinuclear platinum drug to enter clinical trials. It differs from cisplatin in its length and its +4 charge. These differences are believed to enable BBR 3464 to form adducts markedly different in structure to those formed by cisplatin

**Methods** Purified DNA was reacted with BBR 3464 or cisplatin to give an adduct frequency of 1 per kilobase. DNA was also extracted from drug treated human ovarian A2780 cells. Purified DNA was digested using nuclease P1 and DNase 1 and analysed by ionexchange chromatography with elution using NaCl concentration gradients. BBR 3464-DNA was also digested with a higher concentration of the above enzymes (enhanced digestion). Platinum content in fractions were analysed using inductively coupled plasma mass spectrometry (ICP-MS). Purified DNA Samples were analysed using a sensitive competitive ELISA procedure (1) that detects low levels of adducts formed by cisplatin.

**Results** Analysis of cisplatin-DNA adducts using the Mono Q anion-exchange column gave the expected pattern of platinum containing peaks (2). None of the BBR 3464 derived material from enhanced digestion was retained on this column. Analysis using the Macro-prep CM column revealed two main peaks of BBR 3464 derived material that eluted at 6 minutes (1.5M NaCl) and 10.5 minutes (2.6M NaCl). BBR 3464 adducts showed markedly lower immunoreactivity than cisplatin adducts (> 2000 fold). Three to four times the levels of adducts were detected on extracted DNA treated with BBR 3464 as compared to cisplatin at equivalent drug doses.

**Conclusion** The chromatographic behaviour and immunological properties of DNA adducts formed by BBR 3464 indicate markedly different properties compared to cisplatin adducts. The enhanced digestion needed for BBR 3464-DNA samples may indicate that BBR 3464 is distorting the DNA and restricting access of digestive enzymes. It is anticipated that the strong affinity of BBR 3464 adducts to the cation-exchange column will permit maximum sensitivity for quantification by ICP-MS of adducts in DNA from blood cells collected during a recent phase II trial.

1. Tilby et al 1991. Cancer Research. **51**: 123.
2. Fichtinger-Schepman et al 1985. Biochemistry. **24**: 707.

**P244**

**ORAL DNA VACCINATION FOR ERBB2-EXPRESSING MURINE MAMMARY TUMOUR USING ATTENUATED SALMONELLA *TYPHIMURIUM* .**

Michael A*, Dalgleish A, Pandha H- St. George's Hospital Medical School, Oncology Dept. Cranmer Terrace Lonon SW17 ORF

Attenuated salmonellae have been extensively studied as live vectors for delivery of heterologous antigens. They are safe, highly immunogenic and can elicit long-lasting protective systemic and mucosal immunity. Oral immunization with S. *typhimurium* harboring plasmid DNA has resulted in gene delivery to gut lymph nodes, and induction of immunity to antigens encoded by the plasmid. There are several types of Salmonella mutants currently used for gene delivery. The most common, harbour mutations in aromatic amino acids or purin biosynthesis pathway (e.g. *aroA, aroC, aroD and purA, purE*), deficient production of adenylate cyclase (*cya*) or cyclic AMP receptror protein (*crp*) or mutations affecting the global regulatory system (*phoP/phoQ*).
We are evaluating two new S. *thyphimurium mutants*, SPI2-*sifA* and *ssav*, as gene therapy vectors. *In vitro* gene transfer experiments using the plasmid pIRES2, which contains the Green Fluorescent Protein (GFP) reporter gene cDNA under a eukaryotic promoter have been completed in mouse macrophages and human monocyte cell lines-J7742 and U937 respectively. The expression of GFP using fluorescent microscope, was detected in 2% of cells. The same protocol was applied to primary mouse macrophages where expression of GFP was seen in up to 60% of cells.
We plan to use SPI2 mutants as gene therapy vectors using an erbB2 DNA vaccine in mouse mammary tumour model. We have cloned truncated versions of the erbB2 genes representing peptide epitopes for the intracelleular and extracelluelr domains of the erbB2 oncoprotein. We will use S. *typhimurium* to deliver these plasmid constructs as DNA vaccines by the intraperitoneal and oral routes, and compare the efficacy and immunogenicity of this approach with intramuscular delivery of the same constructs.
All in vivo preclinical studies comply with UKCCR " Guidelines for the Welfare of Animals in Experimental Neoplasia."

**P245**

***IN VITRO* ANTI-TUMOUR AND ANTI-ANGIOGENIC ACTIVITY OF AW464 (NSC 706704), A NOVEL QUINOL**

A Mukherjee*[1], SG Martin[1], TD Bradshaw[2], AD Westwell[2], MFG Stevens[2], J Carmichael[1]
Cancer Research UK Academic Unit of Oncology[1], University of Nottingham, Nottingham, UK, Cancer Research Laboratories[2], University of Nottingham, Nottingham, UK

**Introduction/Aims**: AW464 (NSC 706704) is a novel benzothiazole substituted quinol compound with potent and selective activity concentrated in certain colon and renal cancer cell lines. The mechanism of action of AW464 however is not well understood. COMPARE analysis suggested possible interaction with thioredoxin/ thioredoxin reductase signalling. It is unknown whether it is likely to have any significant anti-angiogenic activity. In this study, the effects of AW464 were compared with those of Paclitaxel, which in addition to having direct tumour cell toxicity, also has a proven anti-angiogenic action (Belotti D et al, 1996).

**Materials and Methods***: In vitro* experiments were performed on HT29, SW620 and SW480 colorectal cancer cell lines and on human umbilical vein endothelial cells (HUVEC). The effects of both drugs on cell growth were assessed by the MTS assay and growth curves. Effects on HUVEC differentiation were assessed by the Matrigel assay. In some experiments, HUVEC were exposed to tumour conditioned media to mimic the tumour environment.

**Results**: The MTS assay and growth curve experiments demonstrated that AW464 exerted anti-proliferative effects on tumour cell lines as well as HUVEC at concentrations between $10^{-5}$M and $10^{-7}$M. This activity was comparable with Paclitaxel. HUVEC proliferation was significantly inhibited by exposure to tumour conditioned medium alone. Addition of AW464 further suppressed proliferation slightly. Using the Matrigel assay, endothelial tube formation was partly inhibited within 24-48 hours by AW464 although this effect was less pronounced than with Paclitaxel. We

 © 2002 Cancer Research UK

conclude that AW464 may have an effect on endothelial cell proliferation and differentiation in addition to its tumour cell toxicity.

**Ref.** Belotti D et al (1996) Clinical Cancer Research 2: 1843-1849

**P246**

**THE HORSERADISH PEROXIDASE/INDOLE-3-ACETIC ACID COMBINATION FOR ENZYME PRODRUG THERAPY: RROM *IN VITRO* TO *IN VIVO*.**

J. Tupper*, O. Greco, M.R.L. Stratford, G.M. Tozer, P. Wardman, G.U. Dachs.
Gray Cancer Institute, PO Box 100, Mount Vernon Hospital, Northwood, Middlesex, HA6 2JR, UK.

Cancer treatment is the main application of gene therapy, initially devised to correct inherited monogenic disorders. Gene directed enzyme prodrug therapy (GDEPT) involves the transfer of a gene encoding an enzyme to cells, and the subsequent administration of a prodrug, which is converted to a cytotoxin by the enzyme. Several GDEPT systems have been devised, including HSV thymidine kinase/gancyclovir and cytosine deaminase/5-fluorocytosine. A recently developed system is the horseradish peroxidase/indole-3-acetic acid (HRP/IAA) combination (Greco *et al*., 2000, Cancer Gene Ther. 7, 1414). When expressed in mammalian cells *in vitro*, the plant enzyme HRP is able to convert IAA, a plant hormone, to a cytotoxin.
In order to evaluate this combination further, we utilised the nasopharyngeal squamous cell carcinoma cell line FaDu, transfected with the HRP gene, in tissue culture, in tumour spheroids and in a xenograft tumour model.
We show that HRP expression is able to selectively sensitise FaDu cell monolayers to IAA and 2 of its derivatives, 5bromoindole-3-acetic acid and 1methylindole acetic acid, after 4 and 24 hour exposure. 5bromoindole-3-acetic acid was active at lower concentrations than IAA, with 2-log cell kill at 1 mM in HRP transfectants.
As an intermediate between *in vitro* monolayers and *in vivo* experimental tumours, a 3-dimensional multitumour cell spheroidal model has been developed. IAA and its derivatives were also active in this more complex *in vitro* system.
Xenografted tumours grown from HRP-transfected FaDu cells were shown to retain enzyme activity *in vivo*. Initial pharmacokinetic studies with a single bolus intraperitoneal injection of IAA indicate first order kinetics. With a non-toxic 250 mg/kg dose, plasma, liver and skeletal muscle concentrations peaked 10 minutes after administration. In the tumour, concentrations reached 1 mM, and remained above 0.5 mM for 50 minutes.
These data provide promise for the ongoing evaluation of the HRP/IAA combination for GDEPT.
This work was funded by Cancer Research UK.

**P247**

**DENDRITIC CELL IMMUNOTHERAPY FOR METASTATIC PROSTATE CANCER USING CRYOPRESERVED DENDRITIC CELLS PULSED WITH ALLOGENEIC TUMOUR LYSATE.**

John RJ*, Hutchinson JA, Pandha HS and Dalgleish AG.
Department of Oncology, St George's Hospital Medical School, Cranmer Terrace, London, SW17 ORE, United Kingdom

Immunotherapy as a treatment modality for hormone-refractory prostate cancer (HRPC) has shown promise in recent trials using whole cell vaccines and dendritic cell (DC) approaches. However, DC-based strategies have been limited by the need for repeated venesection or leukapheresis to provide sequential supplies of DCs for modification and vaccination. We have developed a method of culturing then cryopreserving monocyte-derived DCs after pulsing with irradiated allogeneic prostate cancer cell lysate. The freeze-thaw process resulted in minimal loss of cells, increased DC maturation and thus enhanced antigen-presenting capability. This method has provided numerous identical aliquots of DCs; one aliquot is thawed, tested for phenotype, function and microbial contamination prior to administration. In this way we ensured patients received identical sequential vaccines. We have completed a phase I/II, local research ethics committee approved clinical trial of 14 patients with metastatic HRPC who received $1 \times 10^6$ DCs intradermally or intranodally at 2 weekly intervals for 3 months then monthly. Keyhole limpet haemocyanin (KLH) was used as the adjuvant. This protocol was safe and non-toxic with only 2 reports of slight fever. Initial results indicate a PSA response in 4 patients, 2 with an arrest in PSA levels over 4 months and 2 showing reduction in PSA velocity over the same time period. All patients showed a positive delayed-type hypersensitivity test (DTH) prior to commencing treatment confirming their immunocompetence. We intend to assess response to vaccine clinically, radiologically, DTH response to intradermal injection of the tumour lysate, ELISPOT, cell-specific cytokine production using CBA array and proliferative lymphocyte responses to vaccine DCs or recall antigens.

**P248**

**VACCINATION WITH NAKED DNA ENCODING MURINE FLT3 LIGAND DOES NOT CONFURE TUMOUR PROTECTION SEEN WITH RECOMBINANT PROTEIN.**

Matthew Turner*, Hardev Pandha and Angus G. Dalgleish
Division of Oncology, St George's Hospital Medical School, Tooting, London, SW17 ORE, UK

Flt3 ligand is a novel haematopoietic growth factor. Its administration has been shown to increase dendritic cell and natural killer cell numbers and activity in vivo by activating and expanding Dendritic Cell and Natural Killer cell progenitors. Previous studies used the recombinant protein have shown increased tumour rejection and survival in melanoma and lymphoma. However, this is of limited availability and high cost. The use of Polynucleotide DNA using cytokine cDNA cloned into eukaryotic expression vectors has shown efficacy as a vaccine in infectious disease and cancer models. Plasmid DNA expressing the extracellular domain of murine flt3 ligand was produced using a standard DNA extraction kit. Its expression was confirmed by COS cells transfection. We studied the effect of vaccinating with flt3 ligand alone, or in combination with the melanoma antigens TRP1 and TRP2. The cytokine GMCSF was also included in the vaccination protocol. We were unable to reproduce the results demonstrated using the recombinant flt3 ligand with no significant survival or immune responces. Combination of flt3 ligand with antigen did confer a slight protection. GMCSF did produce a survival advantage that appeared independent of tumour antigen.
We are unaware of any published studies using flt3 ligand as a DNA vaccine prior to this.
All in vivo work was carried out in strict accordance with Home Office and Institutional Guidelines

**P249**

### ABSTRACT WITHDRAWN

**P250**

**STRATEGIES TO IMPROVE MURINE MELANOMA ALLOGENEIC VACCINE USING CYTOKINE GENE TRANSFECTION**

Ward, S.J.*[1], Halanek, N.[2], Birchall, L.J.[2], Desel, C.[1], Russell, N.[1], Whelan, M.[1], Dalgleish, A.G.[2]
Onyvax Ltd. [1] and Dept. of Oncology [2], St George's Hospital Medical School, Cranmer Terrace, Tooting, London, U.K., SW17 ORE

Syngeneic tumour cell vaccines are difficult to produce, due to the inherent problems associated with bespoke vaccine production. Stable, well characterised allogeneic tumour cell vaccines containing cross-reactive tumour antigens circumvent these problems, and are an attractive proposition. Using the B16-F10 murine melanoma system, we have shown vaccination with irradiated, allogeneic tumour cells to protect from challenge with live, syngeneic tumour. The introduction of cytokine encoding sequences into syngeneic vaccine cells has been shown to increase vaccine efficacy by several groups (Dranoff, Jaffee, Lazenby *et al*., 1993, PNAS USA, 90:3539). In this study, we introduced the following murine cytokine sequences into B16-F10 cells using the pLXIN (Clontech) retroviral system; GM-CSF, IL-2, IL-4, IL-7. Single clones were selected, rendered replication incompetent by

© 2002 Cancer Research UK

irradiation, and used to immunise both syngeneic (C57BL/6j) and allogeneic (C3H/HeN) female mice sub cutaneously. Following 2 doses of vaccine, mice were challenged on the opposing flank with a tumourigenic dose of syngeneic tumour. Immunisation with allogeneic vaccine expressing cytokine did not confer any real survival benefit when compared to the wild type vaccine. However, transfection with IL-2 did produce a small, but significant, increase in survival time. In contrast, only IL-4 transfection increased the efficacy of wild type vaccine within the syngeneic mice. Splenocytes from allogeneic immunised mice were able to produce significant levels of CTL killing specific for the syngeneic tumour challenge cells. In addition, cytokines such as TNF-alpha, IFN-gamma, IL-2 and IL-5 were also induced by these splenocytes following *in vitro* incubation with both syngeneic whole tumour cells and lysate. Thus, vaccination with allogeneic cells induces a cellular immune response which can cross-react and protect against challenge with syngeneic tumour. The potency of the host's anti-allogeneic response appears to be such that the introduction of additional cytokine genes into the vaccine does not readily improve vaccine efficacy.

**P251**

**DETECTION OF THYMIDYLATE SYNTHASE (TS) INHIBITION**

K.Yau, P. Price, & E.O. Aboagye*, Cancer Research UK PET Oncology Group, Imperial College School of Medicine, MRC Cylotron Unit, Hammersmith Hospital, Du Cane Road, London W12 0NN.

The aim of this study is to validate the use of radiolabelled thymidine ([$^3$H]TdR) uptake as a measure of TS inhibition. The working hypothesis is that TS inhibition causes a depletion in thymidine monophosphate and to overcome this blockade, cells undergoing DNA synthesis will increase the amount of labelled thymidine taken up via the salvage pathway.

RIF-1 cells growing in culture showed an increase in [$^3$H]TdR uptake at 0, 2 and 6 h after a 2-h treatment with 5-fluorouracil (5-FU; 1-100 μg/ml). A decrease in [$^3$H]TdR uptake was observed at 24 and 48 h. Both effects were dose dependent but whereas the maximum increase in [$^3$H]TdR was only 25 ± 2% of controls, the decrease in [$^3$H]TdR uptake at 48 h for instance was >95% at high concentrations. A similar effect was seen in HT29 cells (slower growing) treated with 5-FU, although the decrease in [$^3$H]TdR at the latter time points was less marked. In contrast, treatment of the cells with cisplatin (non-TS inhibitor; negative control) led to a decrease in [$^3$H]TdR uptake at 2, 6, 24 and 48 h. To further confirm that the early increase in [$^3$H]TdR uptake was dependent on TS inhibition, similar studies with the non-classical TS inhibitor AG337 were performed. In RIF-1 cells, AG337 treatment (0.05-5 μg/ml; continuous exposure) resulted in a large increase in [$^3$H]TdR uptake (up to 152 ± 5% of controls at 2 h). The increase in [$^3$H]TdR peaked at 2 h, was still high at 6 h but as with 5-FU, decreased at 24 and 48 h. In all cases MTT assays were performed in parallel with the [$^3$H]TdR uptake studies. These studies showed that the early increase in [$^3$H]TdR uptake observed was not due to an increase in viable cell number. Although a decrease in formazan production was observed in all cases where there was a decrease in [$^3$H]TdR uptake, this was less pronounced compared to the decrease in [$^3$H]TdR uptake. This implies that the decrease in [$^3$H]TdR uptake at the latter time points could be explained in part by a decrease in cell number.

The above studies support the clinical use of [$^{11}$C]thymidine as a non-invasive positron emission tomography probe for imaging TS inhibition in patients and indicate the appropriate timing for such studies. Future experiments will assess whether the changes in [$^3$H]TdR uptake correlate with direct measures of TS inhibition.

**P252**

**CONCOMITANT HER2 AND FARNESYLTRANSFERASE BLOCKADE BY R115777 AND TRASTUZUMAB IN PATIENTS WITH ADVANCED CANCER.**

JS de Bono*, G. Schwartz, LA Hammond, L Smith, A. Patnaik, K. Berg, E. Izbicka, EK Rowinsky, AW Tolcher. Institute for Drug Development, 7979 Wurzbach Road, Suite 400, Zeller Building, Cancer Therapy and Research Center, University of Texas Health Science Center, and Brooke Army Medical Center, San Antonio, TX.

**Introduction:** The recombinant human monoclonal antibody trastuzumab targets cellular proliferation by blocking HER2 signaling. However the majority of patients who overexpress HER2 fail to respond to single agent trastuzumab. Farnesyltransferase regulates the post-translational modification of a number of important proteins including the cell signaling protein ras. Farnesyltransferase inhibition can thereby block ras signaling. Concurrent farnesyltransferase and HER2 blockade result in synergistic antitumor activity in HER2 positive cell lines. R115777 is a potent, selective and non-peptidomimetic inhibitor of farnesyltransferase that can inhibit the growth of H-, K-, and N-ras transformed as well as wild-type ras xenograft tumors. It has single agent clinical activity in breast cancer. **Methods:** A phase I study in patients with HER2 positive disease of oral R115777 and trastuzumab was pursued. Trastuzumab doses were fixed at 4 mg/kg IV day 1, then 2 mg/kg IV weekly. R115777 dose levels studied were 200 mg, 300 mg, and 400-mg po bid for 21-days every 28 days. **Results:** Twenty-one patients (8 breast, 5 NSCLC, 4 colon, and 1 each of ovary, pancreas and leiomyosarcoma) have received 78 courses (range: 1-13; median: 4) of R115777 and trastuzumab. Protracted grade 4 neutropenia lasting more than 5 days, fever, and grade 3 thrombocytopenia were dose limiting at an R115777 dose of 400 mg bid. Non-hematologic toxicities have been mild and include nausea/vomiting), headache and fatigue. No clinically significant decrements in LV ejection fraction have been observed. One patient with colorectal cancer has had a partial response. Overall 4 patients have remained on study for more than 8 months (2 colorectal, 1 breast, 1 leiomyosarcoma). Surrogate studies in peripheral blood mononuclear cells have demonstrated inhibition of hDJ chaperone protein farnesylation at all R115777 doses evaluated. R115777 and trastuzumab can be safely administered at full doses for both agents. **Conclusion:** This combined signal transduction inhibition approach warrants further investigation and a Phase II evaluation of this combination in HER2 positive breast cancer patients is now underway.

**P253**

**PHASE I AND PHARMACOKINETIC (PK) STUDY OF THE EPOTHILONE-B ANALOGUE BMS-247550 BY A CONTINUOUS WEEKLY SCHEDULE.**

JS de Bono*, D Hao, LA. Hammond, AW. Tolcher, K. Berg, A Bass, T. Mays, L Smith, R Drengler, EK Rowinsky. Institute for Drug Development, 7979 Wurzbach Road, Suite 400, Zeller Building, Cancer Therapy and Research Center, University of Texas Health Science Center, San Antonio, Texas.

**Introduction:** Epothilone B is a non-taxane microtubule-targeting agent that induces tubulin polymerization and stabilizes microtubules. Similar to paclitaxel epothilone B blocks the cell cycle at the $G_2$-M transition, thereby inducing apoptosis. Epothilone B and its lactam analogue BMS-247550 are active against paclitaxel-resistant tumor cells. **Methods:** Since weekly administration of other anti-microtubule agents has afforded a favorable toxicity profile BMS-247550 was administered continuously once weekly by a 60-minute intravenous infusion utilizing a 28-day regimen. Toxicity endpoints were sought separately in heavily (HP) and minimally pretreated (MP) patients. **Results:** Twenty patients (M:F=9:11) with a median age of 52 years and a median ECOG PS of 1 (6 colorectal; 3 ovarian; 3 NSCLC; 2 each of prostate, melanoma and breast; and 1 each uterus and cervix) have received 49 four-week courses (range 1 to 5; median 2) of BMS-247550. Dose-limiting toxicities include grade 3 fatigue in 1 HP pt at 20 mg/m$^2$, protracted grade 4 neutropenia (>5 days) in 2 HP patients at 30 mg/m$^2$ and neutropenic sepsis in 1 MP patient at 30mg/m$^2$. Neutropenia also precluded weekly treatment on-time in 2 of 3 MP patients at 30 mg/m$^2$ during course 1. One patient with hormone-, mitoxantrone- and paclitaxel-refractory prostate carcinoma had a greater than 50% PSA decrement (from 5100 to 810) with complete resolution of his tumor related bony pain. This patient developed grade 3 peripheral neuropathy and grade 2 fluid retention necessitating his withdrawal from the study after 6 months of treatment. A minor response was observed in a patient with melanoma, and tumor marker decrements were noted in 3 patients with taxane-refractory ovarian carcinoma. Neuropathy, assessed clinically, by nylon monofilament testing and nerve conduction studies, was observed in 6 patients (NCI-CTC grade 1 or 2 in 5; grade 3 in 1) and seen mainly at doses above 20 mg/m$^2$ with repeated dosing. All other drug-related toxicities have been mild to moderate (≤ grade 2) including anemia, fatigue, myalgias, arthralgias, alopecia, anorexia, nausea, diarrhea, vomiting and thrombocytopenia. PK studies indicate dose-proportional

© 2002 Cancer Research UK

kinetics ($C_{max}$ and $AUC_{(0\text{-}Inf)}$) over the dose-range evaluated. Estimated PK parameters: $t_{1/2}$=50.4±42.7 hour; $V_{ss}$=795.4±331.5 L/m$^2$; Cl=23.9±15.2 L/hr/m$^2$. The erythromycin breath test did not predict drug clearance. Accrual continues at 25 mg/m$^2$/wk (HP) and 30 mg/m$^2$/wk (MP) to define safe dose schedules for MP and HP patients. **Conclusion:** This weekly schedule of BMS247,550 has clinical anticancer activity and warrants broad disease-oriented testing in phase II studies

**P254**

**METABOLICALLY ACTIVE CYTOCHROME P450 CYP1B1 IN SOLID TUMOURS: A NOVEL TARGET FOR CHEMOTHERAPEUTIC INTERVENTION.**

Morag C.E. McFadyen[*1], William T. Melvin[2], Graeme I. Murray[1]. Department of Pathology, [2]Department of Molecular and Cellular Biology, University of Aberdeen, Aberdeen, AB25 2ZD UK.

Cytochrome P450 CYP1B1 is a member of a superfamily of haemoproteins which are central to the oxidative metabolism of a wide variety of endogenous and exogenous compounds. Several of these enzymes have an established role in the metabolic bio-transformation of a variety of anti-cancer drugs. We have previously shown that CYP1B1 is a tumour-specific form of cytochrome P450 highly expressed in a variety of malignant tumours localised specifically to tumour cells[1]. In contrast CYP1B1 protein is un-detectable in corresponding normal tissue. Recently we have identified several anti-cancer drugs that are substrates for CYP1B1[2]. Moreover, our *in vitro* studies have shown that the presence of CYP1B1 reduces the efficacy of some of these neoplastic agents[3]. Cytochrome P450 enzymes require the presence of cytochrome P450 reductase (CPR) for optimal metabolic activity. Since CYP1B1 metabolises anti-cancer drugs in tumour cells it is important to determine the level of active CYP1B1 and CPR in tumours. In this study we demonstrated both CYP1B1 and CPR activity in the microsomal fraction of ovarian (n=9) and kidney tumours (n=24). The Ortho-deethylation of ethoxyresorufin to resorufin was used to measure CYP1B1 activity. We have found CYP1B1 activity in the 100,00g fraction in both ovarian and kidney tumours (100-800 fmol/min/mg of protein). Co-incubation with the CYP1B1 inhibitor alpha-naphthoflavone inhibited this activity. No CYP1B1 activity was observed in any of the normal kidney samples examined (n=8). CPR activity was determined spectrophotometrically by measuring the reduction of cytochrome c at 550nM. CPR activity was detected in all normal and tumour samples (0.04-3nmol/min/mg protein). The presence of CYP1B1 which is capable of metabolising anti-cancer drugs and which is active only in tumour cells highlights a novel target for chemotherapeutic intervention.

References

1. Murray GI, Melvin WT, Greenlee WF, Burke MD. 2001 Annu Rev Pharmacol Toxicol 41: 297.
2. Rochat B, Morsman JM, Murray GI, Figg WD, McLeod HL. 2001 J Pharmacol Exp Ther 296: 537.
3. McFadyen MC, McLeod HL, Jackson FC, Melvin WT, Doehmer J, Murray GI. 2001 Biochem Pharmacol 62: 207.

Acknowledgement: This research was funded by Cancer Research UK and the Gray Fund.

**P255**

**HIGH THROUGHPUT PHARMACOKINETICS OF NOVEL DNA-DEPENDENT PROTEIN KINASE INHIBITORS,**

NF Smith[1]*, FI Raynaud[1], RJ Griffin[2], IR Hardcastle[2], L Rigoreau[2], ML Stockley[2], JJ Leahy[2], BT Golding[2], N Martin[3], GCM Smith[3], and P Workman[1].
[1]CRC Centre for Cancer Therapeutics, Institute of Cancer Research, 15 Cotswold Road, Sutton, Surrey, SM2 5NG, UK. [2]Department of Chemistry, The University of Newcastle upon Tyne, Newcastle upon Tyne, NE1 7RU, UK [3]KuDOS Pharmaceuticals Limited, 372 Cambridge Science Park, Milton Road, Cambridge CB4 0WG, UK

Pharmacokinetic analysis is often a bottleneck in the drug discovery process. The aim of this study was to evaluate cassette dosing, the simultaneous administration of several compounds to a single animal, and *in vitro* metabolic stability screening as methods to predict the pharmacokinetics of a series of novel DNA-dependent protein kinase (DNA-PK) inhibitors in high throughput. DNA-PK is involved in the repair of double strand breaks, thus inhibition of this enzyme may potentiate the effects of radiotherapy or chemotherapy by DNA-damaging agents. NU7053, NU7059, NU7062, NU7119 and NU7163 were administered intravenously to Balb C$^-$ mice alone at 5mg/kg and in combination at 1mg/kg each. Mouse liver microsomal incubations of the five compounds were carried out at a concentration of 10μM for 20 minutes. Plasma and microsomal concentrations were measured by HPLC-MS/MS with multiple reaction monitoring. Pharmacokinetic parameters were evaluated by non-compartmental analysis. The compounds displayed a linear increase in maximum concentration (Cmax) and area under the curve (AUC) with a 5-fold increase in dose from 1mg/kg cassette administration to 5mg/kg single administration. The clearance and half-lives of the compounds were similar following cassette and single dosing. For example, the clearance and half-life of NU7059 following cassette and single dosing was 0.091L/hr and 0.17hr, and 0.081L/hr and 0.16hr respectively. The compound which showed the lowest clearance (0.091L/hr), NU7059, also showed the lowest extent of metabolism (28%). Conversely, NU7053 displayed the highest clearance (0.46L/hr) and metabolism (98%) of the five compounds. The rank order of the compounds from lowest to highest clearance was similar whether they were dosed as single agents (NU7059 > NU7163 > NU7119 > NU7062 > NU7053) or in combination (NU7059 > NU7119 > NU7163 > NU7062 > NU7053). This ranking was maintained in terms of metabolic stability (NU7059 > NU7119 > NU7163 > NU7062 > NU7053). In conclusion, both *in vivo* cassette dosing and *in vitro* metabolic stability screening are suitable methods to evaluate the pharmacokinetics of novel DNA-PK inhibitors. These methods will be used to assess a larger number of compounds from this series in order to increase throughput whilst reducing the number of animals used.

**P256**

**STRUCTURES OF ATYPICAL PLATINUM-DNA ADDUCTS IN CERTAIN RESISTANT LUNG CANCER LINES**

A Azim-Araghi*[1], CJ Ottley[2], DG Pearson[2], MJ Tilby[1]. [1]Cancer Research Unit, The Medical School, University of Newcastle, Newcastle upon Tyne, NE2 4HH, UK, [2]Department of Geological Sciences, Durham University, Durham, UK

**Aims** Cisplatin-DNA adducts, formed in inherently drug resistant adenocarcinoma lung cancer cell line (Mor), and drug sensitive small cell lung cancer line (H69) appear to be markedly different in structure as indicated by differences in immunoreactivity using CP9/19 monoclonal antibody (1). The antibody does not recognise Pt-pApG adducts and the initial aim of the present work was to test the hypothesis that the difference between the cell lines was in the ratio of major DNA adducts.

**Methods** The Pt-DNA adducts present in DNA extracted from the two cell lines exposed to cisplatin were analysed, using anion exchange chromatography (Mono Q), after enzymatic digestion of the Pt-DNA (2). Fractions were collected every 30 seconds, and the total Pt in each fraction was determined, using the sensitive Inductively Coupled Plasma Mass Spectrometry (ICP-MS) technique. This permits analysis of DNA containing relatively low adduct levels (3).

**Results** The high sensitivity of ICP-MS permitted, for the first time, direct analysis of Pt in chromatography fractions from the analysis of DNA extracted from drug treated cells. The ratios of Pt-pGpG to Pt-pApG cross-links were similar in both lines and were consistent with established data (2). However, additional Pt containing peaks were detected compared to those resulting from analysis of reaction products of cisplatin with purified DNA.

**Conclusion** The difference in immunoreactivity, in Pt-DNA adducts of the two cell lines, was not due to differences in relative levels of the major adducts. The additional types of adducts formed in cells do not appear to account for the differences between the cell lines but these need further investigation. The base sequence, where the platinum binds to the DNA is currently being investigated, as these appear to affect the immunoreactivity of Pt-DNA adducts.

1) Tilby et al. 1991, Cancer Res. 51: 123.
2) Fichtinger-Schepman et al. 1985, Biochem. 24: 707.
3) Azim-Araghi et al. 2001, In plasma source mass spectroscopy, by Holland & Tanner, 412.

This research is supported by Cancer Research UK.

**P257**
**TUMOUR PARAMETERS AFFECTED BY COMBRETASTATIN A-4 PHOSPHATE THERAPY IN A HUMAN COLORECTAL XENOGRAFT MODEL IN NUDE MICE**
[1]El-Emir, E., [1]Petrie, I.A., [1]Boxer, G.M., [1]Boden, R., [1]Dearling, J.L.J., [2]Raleigh J.A., [3]Mantavani, A., [1]Green A.J., [1]Begent, R.H.J. and [1]Pedley, R.B. [1]Cancer Research UK Targeting and Imaging Group, Dept of Oncology, Royal Free and University Collage Medical School, Royal Free Campus, London NW3 2PF. [2]University of N.Carolina MS, NC, USA. [3]Instituto di Ricerche Farmacologiche Mario Negri, Milan, Italy.

The effect of the anti-vascular agent combretastatin A-4 phosphate (CA4P) on tumour biology is not fully understood. It has previously been reported that CA4P, a tubulin binding agent, causes vascular shutdown and necrosis in tumours *in vivo*. To further analyse the role of CA4P, we are using a Zeiss microscope with motorised stage and accompanying KS300 image analysis software, to investigate its effect on selected tumour parameters over time using a human colorectal xenograft in nude mice. These include vascular distribution (CD31), perfusion (Hoechst 33342), hypoxia (Pimonidazole), and cell proliferation (BrdU). Mice received either no treatment or a single IP dose of CA4P (200 mg/kg), the latter being sacrificed at either 1 or 24 hours after treatment. Quantitative analysis of relative changes in these parameters following therapy will be discussed.
**Control tumours:** CD31 and Hoechst staining demonstrated perfused blood vessels throughout the whole of the tumour. Similarly, BrdU positive cells were also found in both central and peripheral parts of the tumour. In contrast, pimonidazole staining revealed some areas of hypoxia that tended to be distant from blood vessels and/or surrounding necrosis.
**Treated tumours:** Vascular shutdown studies demonstrated that treatment with a single dose of CA4P resulted in almost complete vascular shutdown 1 hour after treatment. Simultaneous fluorescent staining of both Hoechst and CD31 revealed that although blood vessels were distributed throughout the whole of the tumour, perfused vessels were predominantly in the outer rim with only a few found in the central part of the tumour. Interestingly, BrdU staining revealed that proliferation corresponded to the well perfused outer rim, while only a few nests of cells surrounding blood vessels within the poorly perfused central part of the tumour stained positive for BrdU. These data indicate that a 1 hour treatment with a single dose of CA4P resulted in both vascular shutdown as well as a block in proliferation. Pimonidazole staining, on the other hand, revealed that at this time point, hypoxia was most commonly found in the tumour centre with little or no staining at the outer rim of the tumour. At 24 hours after treatment, when most of the tumour was necrotic, both perfusion and proliferation were restricted to the viable region at the edge of the tumour. Furthermore, pimonidazole staining was also seen in the viable rim, demonstrating hypoxic cells bordering regions of tumour necrosis.
Taken together, the data in this study provide further insight into the mechanisms by which CA4P may exert its effects on tumour cells.
Supported by the Association for International Cancer Research, Cancer Research UK, and Trusthouse Charitable Foundation. CA4P was kindly supplied by OXIGENE.

**P258**
**COMPARISON OF THE CHEMOPREVENTIVE EFFICACY OF CURCUMIN AFTER LONG- OR SHORT-TERM ADMINISTRATION IN MIN/+ MICE.**
Sarah Perkins*, William P Steward and Andreas Gescher.
Department of Oncology, University of Leicester, Leicester LE1 9HN

Curcumin, the major yellow pigment extracted from the spice turmeric, possesses a broad spectrum of chemopreventive efficacy. It has been shown to prevent carcinogen-induced malignancies and premalignancies in rodents. It is also active in the Min/+ mouse, a genetic model of human hereditary familial adenomatous polyposis. The aim of this study was to determine an efficacious dose of curcumin and an optimal time period of administration in the Min/+ mouse. We also determined curcumin levels by HPLC analysis commensurate with potential efficacy. C57BL/6J Min/+ mice were divided into 5 groups (n=10-14 in each). Control mice were kept on a high-protein (RM3) diet, to reflect western-style human diets. Mice received curcumin (0.2% in RM3) for the following time periods: i. perinatally and up to day 30, ii. from day 30 to day 75, iii. from day 75 to day 120, iv. from day 30 to day 120. At 120 days the study was terminated, and small intestinal adenomas were scored. To assess the effect of the high protein basal diet on its own, C57BL/6J Min/+ mice received RM3 or a standard AIN76A diet from day 30 to day 120. Curcumin administered from day 30 to day 120 significantly reduced small intestinal tumour formation by 39% compared to controls ($p<0.05$), whilst administration for shorter periods of time failed to alter tumour burden. The steady state level of curcumin in intestinal mucosa tissue of mice on 0.2% curcumin was 110±23 nmoles/g. Comparison of the basal diets showed that tumour burden is significantly lower when mice are fed a low-protein (AIN76A) diet, decreasing total intestinal adenoma number from 110±8 to 61±3.3 ($p< 0.05$; ± SEM). The results highlight the potential chemopreventive activities of a low-protein diet and of curcumin, and hint at the possible clinical relevance of curcumin as a colorectal cancer chemopreventive agent in humans.

**P259**
***IN VIVO* ANTITUMOUR ACTIVITY AND PHARMACOKINETICS OF NU:UB 31 A ANTHRAQUINONE-PETIDE CONJUGATE**
S. Jackson[1*], J. A. Double[1], P. M. Loadman[1], D. J. Mincher[2], A. Turnbull[2], M. C. Bibby[1]. [1]Cancer Research Unit, University of Bradford, BD7 1DP, UK. [2]Department of Life Sciences, Napier University, Edinburgh, EH10 5DT, UK.
NU:UB 31 is a spacer-linked anthraquinone-L-proline conjugate which belongs to a series of compounds designed to inhibit DNA topoisomerase enzymes. Unlike most topoisomerase inhibitors in current clinical use, NU:UB 31 has been shown *in vitro* to be a dual inhibitor of DNA topoisomerase I and II[1]. The aim of this work was to study the *in vitro* and *in vivo* antitumour activity and pharmacokinetics of the compound. $IC_{50}$ values for the compound calculated against MAC 15A (murine adenocarcinoma) cells using a MTT assay were 5, 8, and 38 μM for 1, 24 and 96-hour exposures respectively. Previous work has shown that treatment with NU:UB 31 at its maximum tolerated dose of 100 mg $kg^{-1}$ led to a significant delay in tumour growth ($P<0.01$)[2]. The activity of doses below the MTD was assessed to look at the therapeutic range of the compound. NU:UB 31 was administered to NMRI mice bearing subcutaneous MAC 15A tumours i.p. (in DMSO/oil) at single doses of 67, and 33 mg $kg^{-1}$ and tumours were measured daily. The growth delay for each dose was 4.3, and 3.2 days respectively. In both cases, treatment resulted in a statistically significant delay in tumour growth ($p<0.01$) compared to controls.
A method was developed to detect and analyse NU:UB 31 in biological samples using LC-MS. Initial work assessed the stability of the compound *in vitro* with NU:UB 31 having $t_{1/2}$ values of 75.1 and 25.7 hours in tissue culture medium and whole murine blood respectively at 37ºC. The compound was very stable in murine plasma with $t_{1/2}$ >100 hours. Pharmacokinetic analysis of NU:UB 31 *in vivo* was assessed in MAC 15A tumour bearing mice. NU:UB 31 was given i.p. at 100mgkg$^{-1}$ and plasma and tumour levels monitored over an 8 hour period. Drug concentration peaked in the plasma after 30 minutes ($C_{max}$ = 12 μg ml$^{-1}$) with a $t_{1/2}$ value of 2.2 hours and AUC of 44.5 μg h ml$^{-1}$. Peak tumour concentrations exceeded those seen in the plasma by approximately 3-fold peaking at 2 hours ($C_{max}$ = 39.5 μg g$^{-1}$) with a $t_{1/2}$ of 6 hours and AUC of 371 μg h g$^{-1}$ (8-fold greater than plasma). $IC_{50}$ values for the compound *in vitro* were converted to CxT values (taking into account the stability of the drug in tissue culture medium) giving values of 18.8, 82, and 176.2μg h ml$^{-1}$ for 1, 24 and 96 hour exposures respectively. The concentration of NU:UB 31 measured in MAC 15A tumours *in vivo* therefore exceeds those of the *in vitro* $IC_{50}$ values. The concentration of NU:UB 31 in tumours and the long half life may go some way to explaining the good anti-tumour activity seen against MAC 15A tumours.
Refs: [1] Gilmour *et al*, Annals of Oncology 9(S2): 62 (1998).
[2] Mincher *et al*, British Journal of Cancer 80(S2), P87: 50 (1999).

**P260**
**EVALUATION OF PHOSPHODIESTERASE I-BASED PROTOCOLS FOR THE DIRECT DETECTION OF TANDEM DAMAGE IN DNA OLIGONUCLEOTIDES.**
KJ Bowman[1*], RC Le Pla[1,2], Y Guichard[1], PB Farmer[2], GDD Jones[1], [1]Department of Oncology, [2]MRC Toxicology Unit, Hodgkin Building, University of Leicester, Leicester, LE1 9HN, UK.

© 2002 Cancer Research UK

Most cytotoxic agents are proposed to exert their deleterious effects via formation of damage in genomic DNA. In addition to 'simple' individual lesions, a number of agents, such as ionising radiation and certain chemotherapeutic drugs, are proposed to generate 'complex' multiply damaged sites (MDS) where several moieties in a local region of the DNA (~10 bp) are damaged. Such complex lesions are proposed to be of heightened biological significance due to the greater challenge they present repair systems.

Given the significance and potential for MDS to occur, it is desirable to have analytical techniques that will allow their direct detection. However, other than indirect measures, such as the measurement of immediate and latent DNA double-strand breaks, there are few analytical techniques that allow direct detection of MDS in DNA.

In the present study we demonstrate the potential of protocols incorporating an exonucleolytic snake venom phosphodiesterase (SVPD, phosphodiesterase I) digestion stage to permit the direct detection of certain tandem damage, in which two lesions are immediately adjacent to each other on the same DNA strand.

A series of oligonucleotides containing either single or pairs of abasic tetrahydrofuran moieties (F) or oxidative thymine glycol lesions ($T^g$) were prepared and digested with SVPD (in the presence of DNase I and alkaline phosphatase (AP)). The digests were then subject to HPLC analysis and the isolated products examined by either $^{32}$P-end-labelling or electrospray mass spectrometry. In addition, a series of oligonucleotides containing either single or pairs of alkylative methylphosphotriester adducts (Me-PTE) were digested with SVPD + NP1 (+DNase I/AP) and directly examined by a $^{32}$P-end-labelling protocol specific for phosphotriester adducts[1]. The unambiguous observation of SVPD-resistant 'trimer' species in the digests of oligonucleotides containing adjacent F, $T^g$ and Me-PTE demonstrates that the SVPD digestion strategy retains some MDS structures and is capable of allowing direct detection of certain tandem damage. Furthermore, in studies to determine the specificity of SVPD in dealing with pairs of lesions on the same strand, it was found mandatory to have the two lesions immediately adjacent to each other in order to generate the trimer species; pairs of lesions separated by as few as one or two normal nucleotides behave principally as single lesions towards SVPD. Although the 5'-phosphodiester linkage adjacent to tandem lesions was highly refractory to SVPD digestion, comparison of peak ratios between SVPD digested oligonucleotides containing single lesions with those containing tandem lesions, following HPLC analysis, suggest that SVPD hydrolysis of the 5'-phosphodiester linkage adjacent to some single lesions can occur.

1. Guichard *et. al.* (2000) *Cancer Research* 60, 1276.

**P261**

**THE SOLUBILISATION AND PURIFICATION OF THE EWS-FLI1 TUMOUR PROTEIN; DETERMINING TERTIARY STRUCTURE TO DEVELOP NOVEL THERAPEUTIC STRATEGIES**

Aneesh Bali*, David Rainey, Tim Ubhi & David Meredith
Molecular Medicine Unit, St James University Hospital, Leeds, LS9 7TF.

**Introduction**

Ewing's sarcoma and primitive neuroectodermal tumour are members of the Ewing's family of tumours. This is consistently associated with chromosomal translocation and functional fusion of the *EWS* gene to transcription factor genes, such as *Fli1* (95% of cases). The resulting chimaeric proteins are believed to contribute to tumour biology by aberrant regulation of gene expression altering controls of cell cycle regulation. These tumour-specific molecular rearrangements are useful as potential therapeutic targets. For this to be effective a detailed understanding of the difference between EWS-Fli1 protein at the level of gene expression is required.

**Methods**

Fli1 and EWS-Fli1 were cloned into the pET32a vector system. Expression of protein was achieved by growth at 37°C until an $A_{600}$ of 0.3 was achieved, at which stage the bacteria were induced with 0.1mM IPTG and grown overnight at 25°C. Cells were harvested, sonicated and pelleted. The supernatant was collected as soluble protein.

Purification involved incubation of nickel NTA-agarose with protein sample for 3 hours. Sample was eluted with 1M Imidazole elution buffer.

Sequencing was conducted with the use of ABI sequencing manual.

**Results**

We have demonstrated expression of Fli1 and EWS-Fli1 proteins by the use of western blot analysis. We have optimised soluble expression of the Fli1 and EWS-Fli1 proteins. 90% purity has been achieved, with the use of affinity chromatography.

Following optimisation of purification, achieved by Ion exchange chromatography, future work would entail analysis of structural similarities between Fli1 and EWS-Fli1.

**Conclusion**

We have managed to achieve high expression of soluble Fli1 and EWS-Fli1. These proteins can therefore be used for detailed three dimensional structural analysis to elucidate differences in DNA:protein binding between the two proteins thus allowing the development of novel therapeutic strategies.

**P262**

**EFFECTS OF DOXORUBICIN AND VINORELBINE *IN VITRO* ON p53 INDUCTION AND PHOSPHORYLATION**

MVCL Appleyard*[1], MA O'Neil[1], AA Liem[1], TR Hupp[2], CR Wolf[3] and AM Thompson[1], [1]Dept. of Surgery & Molecular Oncology, [2]Dept. of Molecular & Cellular Pathology, [3]Biomedical Research Centre, University of Dundee, DD1 9SY, U.K.

**Background** The tumour suppressor p53 is a stress-inducible transcription factor which modulates the expression of genes involved in cell growth or apoptosis. This damage-inducible characteristic of the p53 pathway is linked to the death induced in tumour cells after being exposed to chemotherapeutic drugs or radiation. In order to improve the efficiency of anti-cancer drugs as single agents or in combination, p53 pathway modulation is an obvious choice. In this study we investigated the effects of the anthracycline doxorubicin and the vinca alkaloid vinorelbine on the induction and phosphorylation status of p53 in the breast cancer cell line MCF7.

**Materials and Methods** MCF7 cells were grown in DMEM containing 10% FCS and 1% P/S at 37ºC and 5% $CO_2$. At 70% confluence, medium containing 50 nM doxorubicin or 5 nM Vinorelbine as single agents or a combination of both drugs was added. After 4 and 24 h treatment the cells were harvested by scraping and lysates produced using urea buffer. p53 expression was determined using the antibody DO-12 (specific for p53 core domain). The phosphorylation status at Ser15, Thr18, Ser20 and Ser315 was investigated using phospho-specific monoclonal antibodies.

**Results** Induction of p53 expression was observed after treatment with doxorubicin (single or combination) but not with vinorelbine. A key regulatory step in the control of p53 function implicates the mdm2 ubiquitin-dependent degradation pathway. Ser15 phosphorylation is thought to simultaneously reduce mdm2 binding (and subsequent p53 degradation) and promote interaction of p53 with components of the transcriptional machinery. Ser15 phosphorylation was observed after doxorubicin but not vinorelbine treatment. It has been shown that phosphorylation of p53 at Ser315 primes and stimulates its DNA binding function *in vitro*. Doxorubicin treatment but not vinorelbine resulted in phosphorylation of Ser315. However, when both drugs were added together a decrease in Ser315 phosphorylation was observed compared to the results obtained with addition of doxorubicin on its own.

**Conclusion** This data suggest that chemotherapeutic drug interactions may affect doxorubicin anticancer activity on the p53 pathway.

**P263**

**PHARMACEUTICAL AND BIOLOGICAL PROPERTIES OF THE NOVEL TELOMERASE INHIBITOR RHPS4**

J.C. Cookson*, R.A. Heald, M.F.G. Stevens
Cancer Research Laboratories, School of Pharmaceutical Sciences, University of Nottingham, Nottingham, NG7 2RD, UK.

85-90% of cancers express the enzyme telomerase which is contrastingly absent in benign tumours and most normal somatic cells: telomerase has therefore become a target for cancer treatment. Small molecules capable of stabilising the G-quadruplex structure thought to exist in telomeric repeat sequences, inhibit telomerase. RHPS4 inhibits 50% of telomerase activity at

© 2002 Cancer Research UK

